# Structures of the human glucose-6-phosphate transporter provide insights into its transport cycle and substrate recognition

**DOI:** 10.1371/journal.pbio.3003731

**Published:** 2026-03-30

**Authors:** Wanqin Zhang, Haizhan Jiao, Jingchuan Xue, Jiao Zhou, Yuye Wang, Qi Pan, Yuting Guo, Geshu Zhang, Hongli Hu, Xue Guo

**Affiliations:** 1 Kobilka Institute of Innovative Drug Discovery, Department of Biological Sciences, Division of Biomedical Health Sciences, School of Medicine, The Chinese University of Hong Kong, Shenzhen, Guangdong, China; 2 Guangdong Basic Research Center of Excellence for Ecological Security and Green Development, Key Laboratory for City Cluster Environmental Safety and Green Development of the Ministry of Education, School of Ecology, Environment and Ocean, Guangdong University of Technology, Guangzhou, China; 3 School of Chemistry and Chemical Engineering, Guangdong Pharmaceutical University, Zhongshan, China; University of Zurich, SWITZERLAND

## Abstract

The human glucose-6-phosphate transporter (G6PT/SLC37A4) mediates the translocation of glucose-6-phosphate (G6P) from the cytoplasm into the endoplasmic reticulum, a process essential for glucose production and the maintenance of blood glucose homeostasis between meals. Dysfunction of G6PT causes glycogen storage disease type Ib (GSD-Ib), a severe metabolic disorder characterized by hypoglycemia, hepatomegaly, and neutropenia. Despite its physiological and clinical significance, the structural basis of G6P recognition and the molecular mechanisms underlying GSD-Ib have remained elusive. Here, we present cryo-electron microscopy structures of human G6PT, revealing a monomer in an outward-open state at 3.1 Å and a homodimeric assembly in a face-to-face topology at 3.3 Å. By combining computational modeling of the G6P–G6PT complexes with functional characterization, we have uncovered the key molecular elements that govern the alternating-access mechanism: an electropositive substrate-binding pocket tailored for phosphorylated sugars; conserved aromatic residues that seal the cytosolic gate; and a dynamic inter-domain salt bridge that regulates the conformational transition. Our work provides fundamental insights into the transport cycle of the organophosphate:phosphate antiporter (OPA) family, offers a framework for interpreting GSD-Ib pathology at the molecular level, and establishes a foundation for advancing the mechanistic understanding of the human SLC37 family.

## Abstract 2

The human glucose-6-phosphate transporter (G6PT) mediates the translocation of G6P into the ER, but the structural basis of G6P recognition is unclear. This study solves high-resolution cryo-EM structures of G6PT in both outward-open and dimeric states, providing insights into the mechanistic basis for G6P substrate recognition and transport cycle.

## Introduction

The human glucose-6-phosphate transporter (G6PT, also known as SLC37A4) belongs to the solute carrier 37 (SLC37) family, a group of four transporters (SLC37A1–A4) that reside in the endoplasmic reticulum (ER) membrane [1,2] and share sequence homology with organophosphate:phosphate (Pi) antiporters (OPA) [3–5]. G6PT, SLC37A1, and SLC37A2 are known to mediate the exchange of cytosolic glucose-6-phosphate (G6P) for inorganic Pi [3,6], while the transport properties of SLC37A3 remain to be fully characterized [7,8]. Despite shared transport activity, these transporters exhibit significant sequence divergence, indicating distinct physiological roles.

Within the SLC37 family, G6PT stands out due to its functional coupling with glucose-6-phosphatases (G6Pases), the ER enzymes responsible for hydrolyzing G6P to glucose and Pi [9–11]. In gluconeogenic tissues—liver, kidney, and intestine—the conversion of G6P to glucose is a step critical for maintaining blood glucose levels during fasting [12]. This reaction requires G6PT-mediated import of cytosolic G6P into the ER lumen, providing substrates to G6Pase-α. During catalysis, the luminal Pi generated by G6Pase-α supports G6PT’s transport activity. Deficiencies in either G6PT or G6Pase-α impair blood glucose homeostasis. G6PT also cooperates with the ubiquitously expressed G6Pase-β, forming a functional complex indispensable for normal functions of neutrophils and macrophages [13–16]. Highlighting its fundamental role in human physiology, mutations in G6PT cause glycogen storage disease type Ib (GSD-Ib), a severe metabolic disorder characterized by impaired glucose homeostasis, fasting hypoglycemia, hepatorenal glycogen accumulation, lactic acidemia, hyperlipidemia, and neutropenia with associated immune dysfunction [17–21]. Over 100 mutations in G6PT have been identified in GSD-Ib patients, many of which abolish or impair transport activity [10,22–26]. Yet, though G6PT has long been a subject of intense inquiry in the fields of metabolic disease, biochemistry, and membrane transport, its molecular mechanism has remained elusive due to the lack of a high-resolution structure.

The bacterial homolog of G6PT, glycerol-3-phosphate transporter (GlpT) from *Escherichia coli*, has served as a paradigm for the OPA family. GlpT is an antiporter that couples the import of glycerol-3-phosphate with the efflux of Pi and can also mediate Pi/Pi exchange in reconstituted proteoliposomes [27]. As one of the first major facilitator superfamily (MFS) members to be structurally characterized, the crystal structure of GlpT revealed a canonical MFS-fold in a cytosol-open (inward-open) state, providing foundational insights into the rocker-switch alternating-access mechanism shared by MFS transporters [28]. However, this GlpT structure represents the sole experimental structure for the OPA family. This absence of other major conformations of a transport cycle, along with notable sequence and functional divergence, has hindered a mechanistic understanding of OPA family members, particularly for human G6PT.

## Results

### Structural determination and overall structure of the G6PT monomer

To determine the atomic structure of human G6PT (UniProt: O43826), the protein was overexpressed in HEK293S GnTI^-^ cells and purified in lauryl maltose neopentyl glycol (LMNG) supplemented with cholesteryl hemisuccinate (CHS) (S1A Fig). Due to its relatively small size (~47 kDa) and potential conformational heterogeneity, however, initial single-particle cryo-EM studies on G6PT failed to yield high-resolution reconstructions. To overcome these hurdles, we implemented an optimized computational pipeline centered on five key aspects: (i) isolating of a homogeneous particle subset using seed-guided 2D classification and heterogeneous refinement in CryoSPARC [29]; (ii) increasing the initial and maximum resolutions for ab initio reconstruction in CryoSPARC to 15 Å and 6 Å, respectively, to capture finer structural details in the early stages; (iii) performing 3D auto-refinement in RELION 5.0 with Blush regularization, a deep-learning-based approach specifically designed to improve reconstructions of datasets with low signal-to-noise ratios [30]; (iv) systematically eliminating noncontributory particles from the final stacks with CryoSIEVE [31]; and (v) increasing the extraction box and corresponding mask sizes during the final 3D refinement steps in RELION to optimize signal capture and CTF correction. This integrated workflow ultimately yielded a final reconstruction with an overall resolution of 3.1 Å (S1 Fig and S1 Table). The resulting map confirms a monomeric configuration of human G6PT.

**Fig 1:**
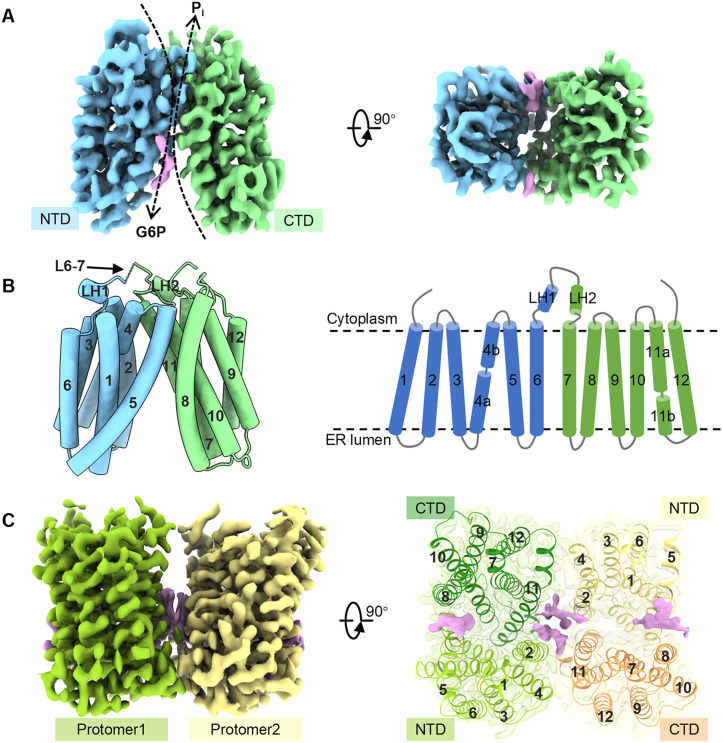
Overall structures of human G6PT in monomeric and dimeric states. (**A**) Cryo-EM density map of the G6PT monomer, viewed parallel to the membrane (left) and from the ER lumen (right). The NTD and CTD are colored blue and green, respectively. Nonprotein densities are shown in plum. (**B**) Overall structure (left) and topology diagram (right) of the G6PT monomer shown parallel to the membrane. The missing region of L6–7 is represented by dashed lines. (**C**) Cryo-EM density map (left) and atomic model (right) of the G6PT homodimer, viewed parallel to the membrane and from the ER lumen. Two protomers are colored yellow-green and khaki, with nonprotein densities highlighted in plum.

G6PT displays a classic MFS fold, with 12 transmembrane helices (TMs) arranged into two pseudosymmetric bundles: the N-terminal domain (NTD; TMs 1–6) and the C-terminal domain (CTD; TMs 7–12) (Fig 1A and 1B). These domains are connected by a long, partially disordered cytosolic loop (L6–7) between TM6 and TM7. The central segment of L6-7 (residues 199–213) is not visible in the density map, suggesting that G6PT lacks the well-ordered intracellular helical bundle characteristic of sugar transporters in the MFS superfamily [32,33] (S3 Fig). The N-terminus of L6-7 (residues 191–194) forms a short helical turn that packs against the cytosolic face of the NTD. Around the kink of the turn, the pathogenic mutation P191L substantially impairs G6P transport [25], pointing out the functional importance of this structural element in possibly stabilizing G6PT’s conformation.

Our structure captures G6PT in an outward-open (ER lumen-open) conformation, in which a tightly packed NTD–CTD interface is formed at the cytosolic side, while the two domains diverge toward the ER lumen. This divergence creates V-shaped openings in the membrane between TM2 and TM11 on one side and TM5 and TM8 on the other side. Viewed perpendicular to the membrane, both crevices are filled with nonprotein densities, presumably lipids or detergents (Fig 1A).

### The dimerization interface of G6PT

In addition to the monomeric form, our cryo-EM analysis resolved a G6PT homodimer at a resolution of 3.3 Å (S2 Fig and S1 Table). The dimer exhibits pseudo-C2 symmetry and each protomer adopts the same outward-open conformation seen in the monomeric reconstruction, allowing for the unambiguous docking of the monomeric model (Fig 1C). The G6PT dimeric assembly is characterized by a face-to-face topology similar to that reported for MCT2 [34], but distinct from the side-by-side arrangements observed in NRT1.1 or MCT8 [35,36]. Dimerization is mediated by symmetric interactions between TM2/TM4 of one protomer and TM11 of the opposing protomer. Notably, the dimer interface contains two prominent nonprotein densities likely representing detergent or lipid molecules, which appear to bridge and stabilize the two subunits.

The physiological relevance of this dimeric state remains elusive. Although the bacterial homolog GlpT functions as a monomer, oligomerization is a documented feature of many MFS transporters. In our structure, the dimer interface incorporates the sites of two GSD-Ib-causing mutations, S54R and S55R (S4A Fig). Previous studies indicate that these variants maintain expression levels comparable to the wild type but exhibit severely impaired transport activity [23]. The finding that the interface mutations result in functional defects rather than protein instability may suggest that the integrity of the dimer interface, or specific residues within it, is important for G6P transport.

However, caution is warranted when interpreting the physiological significance of this dimeric assembly. Recent literature suggests that G6PT oligomerization is highly sensitive to experimental conditions. Specifically, the use of detergents such as LMNG and CHS may facilitate the formation of nonnative oligomers [37,38]. While certain interface mutations have been shown to impair transport [39], they may disrupt the local packing of the transmembrane helices or hinder the conformational transitions required for the transport cycle.

Nevertheless, it remains to be elucidated whether such dimerization reflects a functional status under physiological conditions. Given that the conformation of each protomer in the dimer is identical to the monomeric structure, we have used the monomer for all subsequent mechanistic analyses.

### Translocation pathway

**Fig 2:**
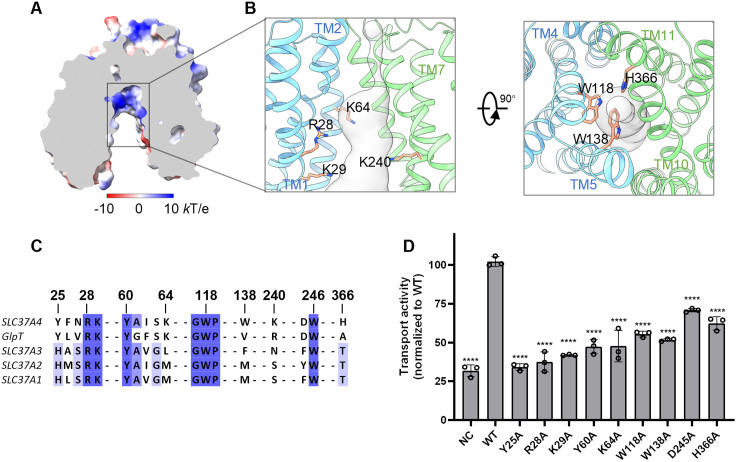
Structure and functional characterization of the G6PT translocation pathway. (**A**) Electrostatic surface of the human G6PT translocation pathway. The surface is colored according to the electrostatic potential (red, −10 *k*T/e; blue, 10 *k*T/e). (**B**) Detailed views of the translocation pathway with key basic residues (left) and aromatic residues (right) lining the cavity shown as sticks. (**C**) The sequence alignment of key residues lining the translocation pathway of human SLC37 members and the bacterial homolog GlpT. Residues are colored according to their level of conservation. (**D**) Transport activities of human G6PT with mutations in the central cavity, normalized to wild type (WT). Data are presented as means ± SD (*n* = 3 replicates). Statistical significance was determined using a one-way ANOVA with Dunnett’s multiple-comparison test. ****P < 0.0001. The data underlying this figure are available in S1 Data.

The outward-open structure of G6PT reveals a large, solvent-accessible cavity open to the ER lumen. A defining feature of this translocation pathway is its electropositive surface located midway through the membrane (Fig 2A), which is ideal for attracting and accommodating negatively charged cargos, such as G6P and inorganic Pi. This positively charged environment is primarily contributed by R28 and K29 on TM1, K64 on TM2, and K240 on TM7 (Fig 2B left). Notably, the ^28^RK motif is a hallmark of the OPA family (Figs 2C and S5. Two lines of evidence underscore the functional necessity of this motif: first, the pathogenic variants R28C and R28H have been reported to severely impair G6P uptake [23]; second, the equivalent ^45^RK motif in the bacterial homolog GlpT is essential for glycerol-3-phosphate transport [40]. To validate these findings in human G6PT, we performed a G6P uptake assay on G6PT variants with single alanine substitutions at the R28 or K29 positions. Both mutants displayed significantly reduced transport activity (Fig 2D), suggesting that the ^28^RK motif acts as a key structural determinant for substrate recognition.

In this outward-open conformation, while the central cavity is accessible from the ER lumen, the translocation pathway is sealed from the cytoplasm by a gate composed of three aromatic residues, W118 on TM4, W138 on TM5, and H366 on TM11 (Fig 2B right). These residues likely play a dual role in stabilizing the cytoplasmic gate and facilitating sugar moiety binding through aromatic stacking or hydrophobic interactions. A key structural element here is the highly conserved ^117^GWP motif, which introduces a helical kink in TM4 that directs the bulky sidechain of W118 into the translocation pathway. The indispensable nature of this residue is demonstrated by the GSD-Ib-causing mutation W118R, which has been reported to abolish transport [23]. Our functional analysis confirms the importance of W118, as its alanine substitution reduces G6P transport activity by 40% (Fig 2D). Furthermore, while W138 and H366 are conserved in vertebrate G6PT homologs but divergent among human SLC37A1–A3 (Figs 2C and S6), mutagenesis analysis shows that both the W138A and H366A substitutions result in substantial activity loss (Fig 2D). Together, these findings highlight the coordinated roles of this aromatic cluster in sealing the cytosolic gate and potentially modulating substrate binding.

### G6P recognition in G6PT

To investigate the mechanism of substrate recognition, we performed cryo-EM studies on G6PT samples pre-incubated with either 50 mM G6P or Pi. However, the resulting reconstructions resembled the apo outward-open configuration and showed no additional ligand density within the central cavity. We then generated G6P-bound G6PT models in both outward- and inward-open conformations using AlphaFold3 [41] and validated the predicted binding modes through molecular dynamics (MD) simulations.

**Fig 3:**
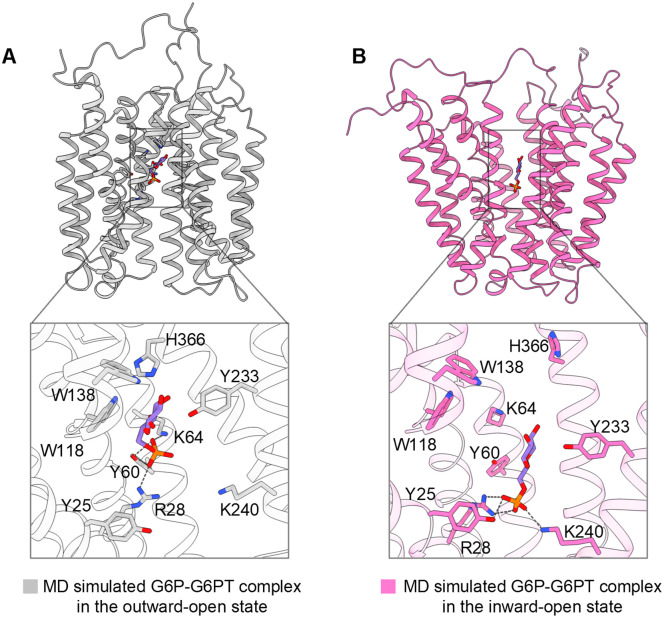
Substrate recognition modes of G6PT revealed by MD simulations. (**A**) Representative binding pose of G6P in the outward-open state. A structural snapshot highlights the interaction details between G6P and G6PT. G6P and interacting residues are shown as sticks; polar interactions (hydrogen bonds and salt bridges) are indicated by dashed lines. (**B**) Representative binding pose of G6P in the inward-open state. Details of the substrate-binding pocket are shown in the same way as in (A).

The predicted outward-open G6P–G6PT complex closely aligns with our experimental apo structure, with the primary difference of a G6P molecule occupying the positively charged cavity (S7A Fig). Across three independent simulations over a 300-ns timescale, the G6P molecule settled into a stable pose at the base of the central cavity. The phosphate group is anchored by R28, Y60, and K64, while the glucose moiety is primarily coordinated by W118, W138, Y233, and H366 (Figs 3A, S7B, and S7C). Conversely, simulations initiated from the inward-open G6P-G6PT model exhibited greater deviations in binding modes, although the putative pocket remained structurally stable (S7D Fig). In two of the three simulations, the G6P phosphate group formed polar interactions with Y25, R28, and K240 (Fig 3B), whereas in the third simulation, it was engaged by R28 and Y60 (S7D Fig). Notably, while the phosphate group of G6P maintains specific contacts with G6PT, the glucose moiety occupies a broader region distal to the positively charged surface of the central cavity. This region appears to provide a hydrophobic environment that accommodates the sugar ring with significant conformational plasticity, as no conserved contacts were observed across the simulations.

Our experimental analysis strongly corroborates these computational findings. Alanine substitutions of key residues predicted to engage G6P, including Y25, R28, and Y60, result in varying degrees of transport defects (Fig 2D). Given that R28 maintains stable interactions with the phosphate group across both major conformational states, and its mutation causes the most profound loss of G6P uptake, we propose that R28 serves as the primary determinant of phosphorylated sugar recognition during the transport cycle.

Comparative sequence analysis further clarifies the substrate specificity within the SLC37 family. While R28 and Y60 are conserved across the OPA family, other cavity-lining residues show significant divergence (Fig 2C). For instance, in SLC37A1–3, the equivalent position of Y25 is substituted with a histidine residue, and the basic residue K64 is replaced by uncharged residues. These variations likely alter the geometry and electrostatic landscape of the binding pocket, fine-tuning substrate specificity and affinity to suit the distinct physiological roles of each transporter. Obtaining experimental structures in substrate-bound states will be essential to validate these predicted binding modes and fully elucidate the mechanisms of substrate selectivity.

### Conformational changes

The cryo-EM structure of human G6PT represents an outward-open conformation. This complements the inward-open state previously observed in the bacterial homolog GlpT (PDB:1PW4) and provides a structural template for elucidating the rocker-switch mechanism of the OPA family. To gain insights into the conformational transition, we compared our experimental structure with an AlphaFold-predicted (AF-predicted) model of G6PT, which adopts a canonical inward-open fold homologous to the GlpT structure.

**Fig 4:**
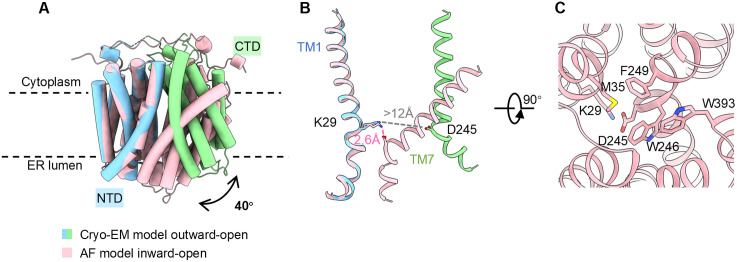
Conformational changes of G6PT. (**A**) Structural comparison of the AF-predicted inward-open model (pink) and the experimental outward-open structure (NTD, blue; CTD, green). The alignment is based on the superimposition of NTDs. (**B**) Interactions between K29 (TM1) and D245 (TM7) during the conformational transition. (**C**) A close-up view of the luminal gate in the inward-open model. Key residues are shown as sticks.

Superposing the NTDs of these two conformational snapshots reveals that the CTD undergoes an ~40° rigid-body rotation relative to the NTD during the transition from the outward-open to the inward-open state (Fig 4A). This movement brings the luminal halves of TM7, TM8, and TM11 of the CTD in close proximity to the corresponding halves of TM1, TM2, and TM5 of the NTD, effectively sealing the central cavity from the ER lumen. An inter-domain salt bridge between K29 (TM1) and D245 (TM7), coordinated with a network of hydrophobic interactions, appears to contribute to the closure of the luminal gate. This salt bridge forms in the inward-open state but breaks in the outward-open structure (Fig 4B). The functional importance of this interaction is supported by our mutational analysis: alanine substitutions of either K29 or D245 reduce transport activity (Fig 2D). This gating mechanism is consistent with molecular dynamics studies of GlpT, which demonstrate that the dynamic formation and breakage of the equivalent salt bridge (K46–D274) facilitates conformational transitions [40]. The gating interface is further reinforced by a cluster of hydrophobic residues—M35, W246, F249, and W393—which likely work in concert with the electrostatic interaction to maintain the integrity of the luminal gate (Fig 4C).

### Missense mutations underlying GSD-Ib

To date, 57 missense mutations have been identified in GSD-Ib, most of which lead to abolished or reduced activity [22,23,25,26]. To understand how these mutations may affect G6PT function, we mapped them onto our structure (S4B Fig). Most mutations are found in TM regions with their sidechains pointing to the interior of the NTD or CTD, suggesting that they probably affect the protein’s folding or stability (S2 Table). For instance, introducing a charged aspartate at G20 on TM1 or at G339 on TM10 has been shown to reduce activity and decrease protein levels [23]. Several of the remaining mutations are located along the translocation pathway. Among these, R28 variants and W118R may directly interfere with substrate binding, while G68R and W138R probably disrupt conformational transitions by destabilizing the NTD–CTD interface.

## Discussion

The mechanism by which G6PT recognizes and translocates G6P has long been a subject of intense investigation. In this study, we determined the cryo-EM structures of human G6PT in an outward-open conformation, elucidated the principles of substrate recognition through MD simulations, and identified key conformational dynamics by comparative structural analysis. These findings provide a structural blueprint that improves our understanding of the molecular basis of G6PT function and the pathology of GSD-Ib.

G6PT adopts a canonical MFS fold characterized by an electropositive central cavity, which distinguishes it from neutral sugar transporters within the MFS superfamily, such as GLUTs. This cationic environment is established by a cluster of four basic residues—R28, K29, K64, and K240—which collectively attract and coordinate the negatively charged phosphate moiety of G6P. Our MD simulations of G6P–G6PT complexes, supported by functional mutagenesis, confirm the pivotal roles of these residues. R28 maintains a stable interaction with the phosphate group across both outward- and inward-open states. This interaction is reinforced by Y60 and K64 in the outward-open state, while it is facilitated by K240 in the inward-open state. Our simulations further reveal that G6P exhibits greater stability in the outward-open configuration, whereas the inward-open state allows for more mobile binding poses.

During the preparation of this manuscript, several independent studies reported structures of G6PT in apo, substrate-bound, and inhibitor-bound states [37–39,42]. These studies used diverse approaches, including nanodisc reconstitution and fusion-tag nanobodies, to capture various states of the transporter. Interestingly, structural comparisons reveal a range of binding poses of G6P in both the inward-open and outward-open states. This experimental diversity is consistent with the plasticity observed in our MD simulations (S8 Fig), suggesting that G6PT uses a fluid binding pocket to facilitate substrate capture and release. The convergence of our computational findings with these independent structural snapshots cross-validates the role of key residues such as R28, Y60, K64, W118, W138, Y233, and H366 in defining the translocation pathway. Together, these complementary datasets capture critical intermediates in the loading and release of G6P, offering a comprehensive view of the G6PT transport cycle.

While R28 is universal across prokaryotic and eukaryotic OPA members, other basic residues in the central cavity show strategic divergence. For instance, K64 (TM2) is conserved between G6PT and its bacterial homologs like GlpT but is absent in human SLC37A1–3, indicating a potential point of functional divergence. Moreover, the position of K240 on TM7 varies: it is an arginine in bacterial transporters, whereas in human SLC37A1–3, the equivalent lysine is shifted by approximately one helical turn. These variations may alter the geometry and electrostatic landscape of the substrate-binding pocket to meet the specific physiological demands of each SLC37 member.

Beyond substrate recognition, the conserved ^28^RK motif appears to play an additional role in the luminal gate formation. While K29 forms a stable salt bridge with D245 in the predicted inward-open state, our outward-open structure shows these two residues separated by over 12 Å (Fig 4B). This observation provides structural evidence for the gating mechanism initially proposed for GlpT, in which breaking an equivalent salt bridge (K46–D274) upon substrate binding facilitates the conformational transition [40]. Given that the corresponding aspartate (D245) is absent in SLC37A1–3, this gating mechanism may distinguish G6PT from other human SLC37 family members. Such mechanistic variation reflects an evolutionary split that clusters SLC37A4 with its bacterial ancestors, which establishes a foundation for resolving the functional diversity within the human SLC37 family.

Finally, our cryo-EM analysis identified nonprotein densities within the lateral openings at the NTD–CTD interface (Fig 1A and 1C). In the dimeric assembly, these densities appear to act as a “molecular glue” stabilizing the dimer interface, an observation that aligns with recent structural findings [37,39]. In the monomeric state, these unknown molecules effectively shield the transmembrane core from the surrounding lipid bilayer. These wide lateral portals are structurally reminiscent of the substrate entry routes found in MFS lipid transporters such as MFSD2A [43]. This structural similarity raises the intriguing possibility that specific lipids in the native ER membrane occupy these openings to modulate the transporter’s stability or conformational transitions during the transport cycle. Elucidating the functional impact of these lipid–protein interactions will be essential for a comprehensive understanding of G6PT activity within its physiological environment.

## Materials and methods

### Expression and purification of G6PT

The full-length gene encoding human G6PT was codon-optimized and cloned into the pEGBacMam vector with an N-terminal Flag tag and a C-terminal EGFP. Recombinant G6PT protein was expressed in HEK293S GnTI^−^ cells using the BacMam system. Briefly, upon reaching a density of 2 × 10^6^ cells/mL, cells were transduced with P_2_ baculoviruses at a volume ratio of 1:50 (v/v). The cells were then harvested at 48 hours post-infection and stored at −80 °C until further use.

For purification, cell pellets were resuspended and homogenized in a solubilization buffer containing 20 mM HEPES pH 7.5, 150 mM NaCl, 1% (w/v) lauryl maltose neopentyl glycol (LMNG, Anatrace), 0.1% (w/v) cholesteryl hemisuccinate (CHS, Anatrace), and a protease inhibitor cocktail. The lysate was clarified by centrifugation at 20,000 rpm for 45 min. The resulting supernatant was loaded onto anti-DYKDDDDK affinity resin and incubated for 2 hours at 4 °C. The resin was then washed with 20 column volumes of a wash buffer containing 20 mM HEPES pH 7.5, 150 mM NaCl, 0.1% (w/v) LMNG, 0.01% (w/v) CHS. The protein was subsequently eluted with an elution buffer containing 20 mM HEPES pH 7.5, 150 mM NaCl, 0.01% (w/v) LMNG, 0.001% (w/v) CHS, and 0.25 mg/mL Flag peptide. The eluate was concentrated using a 100-kDa cutoff concentrator and further purified by size-exclusion chromatography (SEC) on a Superdex 200 Increase 10/300 GL column (GE Healthcare) using SEC buffer [20 mM HEPES pH 7.5, 150 mM NaCl, 0.005% (w/v) LMNG, 0.0005% (w/v) CHS]. Peak fractions were pooled and stored at −80 °C.

### Cryo-EM sample preparation and data acquisition

For cryo-EM grid preparation, 3 μL of the purified G6PT protein was applied to glow-discharged (15 W for 45 s) gold grids with an amorphous alloy film (UltraAuFoil Au 300-mesh R1.2/1.3). The grids were blotted for 3.5 s and then plunge-vitrified into liquid ethane at 4 °C under 100% humidity using a Vitrobot Mark IV (Thermo Scientific, USA). Cryo-EM data were acquired on a 300 kV Titan Krios Gi3 (Thermo Fisher Scientific) equipped with a Gatan K3 Summit detector. Automated data collection was performed using SerialEM 4.1 software. Raw movies were recorded at a nominal magnification of 105,000×, resulting in a pixel size of 0.425 Å. Each movie stack consisted of 50 frames collected over 2.9 s, with a total electron dose of approximately 50–54 *e*^*−*^*/Å*^*2*^. The defocus range was set between −1.5 and −2.0 μm.

### Cryo-EM data processing and 3D reconstruction

For the G6PT monomer reconstruction, a dataset of 13,581 movie stacks was subjected to motion correction using MotionCorr2 in RELION 5.0 [44]. The micrographs were then imported into CryoSPARC [45] for patch contrast transfer function (CTF) estimation. Particles were picked and extracted for multiple rounds of 2D classification. To eliminate low-quality particles and resolve conformational heterogeneity, iterative rounds of ab initio reconstruction and heterogeneous refinement were performed. A subset of 1,691,620 particles was re-extracted in RELION at a pixel size of 0.85 Å for 3D classification. A 3D class containing 283,090 particles and clearly exhibiting 12 TMs was isolated for 3D auto-refinement. The resulting particle stack was refined further using CryoSIEVE to improve the map quality. After several rounds of 3D auto-refinement, post-processing, and polishing in RELION, the final monomeric reconstruction reached an overall resolution of 3.1 Å.

The G6PT dimer was processed using a similar computational workflow. A total of 4,833 movies were motion-corrected and subjected to Patch CTF estimation, as described above. Seed-guided 2D classification, ab initio reconstruction, and heterogeneous refinement were then performed in CryoSPARC. A subset of 1,044,270 particles was re-extracted in RELION at a pixel size of 0.85 Å, and two rounds of 3D classification reduced the particle number to 371,421. This number was then refined to 152,134 by CryoSIEVE. After CTF refinement and particle polishing, the final particle set was imported back into CryoSPARC for nonuniform and local refinement. This yielded a final map at a resolution of 3.3 Å.

### Model building, refinement, and validation

The initial G6PT monomer model was generated by rigid-body fitting the NTD (residues 6–198) and CTD (residues 214–422) from an AlphaFold-predicted structure into the 3.1 Å cryo-EM map with ChimeraX [46]. The model was then manually adjusted in Coot [47] and refined in PHENIX [48]. For the G6PT homodimer, the refined monomeric coordinates were docked into the 3.3 Å dimeric density map. Characteristic nonprotein densities observed at the subunit interfaces were tentatively modeled as LMNG molecules (S2C Fig). The final models were validated using MolProbity within PHENIX to ensure optimal stereochemistry and map-to-model agreement.

The translocation pathway was calculated and analyzed using MOLE [49]. Density maps and structural figures were generated using ChimeraX. Sequence alignments were performed using ClustalW [50] and visualized in Jalview [51].

### G6P uptake assay

The G6P transport activity was evaluated using a cell-based uptake assay as described in a recent protocol [42]. Full-length human G6PT (WT and mutants) and human G6PC1 (WT; UniProt No. P35575) were cloned into the pEGBacMam vector, and recombinant baculoviruses were produced using the BacMam system. Expi293F cells were co-infected with P_2_ baculoviruses encoding G6PC1 and the respective G6PT (WT or mutant) and harvested at 24 hours post-infection for functional analysis.

For each experimental replicate, 8 × 10^6^ cells (or 4 mL of culture) were harvested and washed once with 2 mL of 1× Hank’s balanced salt solution (HBSS buffer) containing 2% FBS. The cells were permeabilized with 1 mL of 1× HBSS buffer containing 10 μM digitonin for 10 min at 37 °C. The treated cells were subsequently washed with 500 μL of 1× HBSS buffer and collected by centrifugation at 1,000 rpm for 3 min. The transport reaction was initiated by adding 1 mM [^13^C]-G6P to the 200 μL reaction system and then incubated at 37 °C for 10 min. The reaction was terminated by adding ice-cold 1× HBSS buffer, followed by two rapid wash steps to remove extracellular substrates. Intracellular metabolites were extracted with 400 μL of 80% methanol at −80 °C overnight. The resulting supernatant was analyzed by mass spectrometry to quantify the internalized [^13^C]-G6P. All transport activities were normalized to the total protein concentration determined by a BCA assay. Noninfected cells were processed in parallel to serve as a negative control.

### G6P-G6PT modeling via AlphaFold3

Structural models of the human G6PT in complex with G6P were generated using AlphaFold3. Two distinct configurations were modeled: (1) G6PT with G6P alone and (2) G6PT with both G6P and Pi. The amino acid sequence of G6PT and the SMILES strings for the ligands were converted to JSON input files. Predictions were carried out on a GPU-accelerated computational node using varying random seeds to ensure broad sampling. The resulting models were manually evaluated based on conformational consistency and confidence metrics, specifically the interface predicted Template Modeling (ipTM) scores. Notably, all G6P–Pi–G6PT models converged to an inward-open state. In contrast, four out of five G6P–G6PT models adopted an outward-open conformation. The configurations with the highest confidence scores—the outward-open G6P-G6PT complex (ipTM = 0.86) and the inward-open G6P–Pi–G6PT complex (ipTM = 0.77)—were selected as starting structures for subsequent MD simulations. Pi was found to be dissociated from the transporter core in the G6P–Pi–G6PT models; therefore, it was omitted from the final model used for structural analysis and interpretation.

### MD simulation

Simulations were initiated from high-confidence AlphaFold3 models of G6PT in both the G6P-bound outward-open and inward-open conformations. Each system was oriented using the PPM server [52] and embedded in a pre-equilibrated lipid bilayer using the CHARMM-GUI [53,54] membrane builder. To ensure sampling robustness, three independent replicates were performed for each conformational state.

All simulations were executed using the GROMACS package [55], employing the CHARMM36 force field for the protein [56], G6P, lipids, and ions, and the TIP3P model for water molecules [57]. Constant temperature (300 K) and pressure were maintained by a velocity-rescaling thermostat and a semi-isotropic C-rescale barostat, respectively. Long-range electrostatic interactions were calculated using the Particle Mesh Ewald (PME) method. Van der Waals interactions were computed with a 12 Å cutoff, using a force-switching function applied between 10 and 12 Å.

Following a multi-step equilibration protocol to relax lipids and solvents, three independent 300-ns production runs were performed under the NPT ensemble. A time step of 1 fs was used during equilibration, and 2 fs was used for production runs. Trajectory analysis was performed on the final 100 ns of each production simulation. Binding free energies for the G6P–G6PT interactions were calculated using the MM/GBSA method [58] based on the final 100 ns of the production trajectories.

## Supporting information

S1 Fig Purification and cryo-EM reconstruction of the G6PT monomer. (**A**) Representative size-exclusion chromatography (SEC) profile and corresponding SDS-PAGE analysis of purified G6PT. (**B**) Cryo-EM data processing workflow for the G6PT monomer. (**C**) Fourier shell correlation (FSC) curve for the final reconstruction (top) and the particle orientation distribution (bottom) as evaluated by RELION 5.0. (**D**) Cryo-EM density maps for 12 TMs of G6PT.
(TIF)

S2 Fig Cryo‑EM reconstruction of the G6PT dimer. (**A**) Cryo-EM data processing workflow for the G6PT dimer. (**B**) FSC curves for the final reconstruction (left) and the particle orientation distribution (right) as evaluated by CryoSPARC. (**C**) Structural models of LMNG fitted into nonprotein densities at the dimer interface.
(TIF)

S3 Fig Structural comparison of human G6PT and GLUT3. The G6PT cryo-EM structure with the L6–7 highlighted in orange and the GLUT3 structure (PDB: 4ZWC) with the intracellular helical (ICH) domain between TM6 and TM7 highlighted in green. G6PT has an L6–7 loop that lacks the triple-helix domain characteristic of GLUTs. The GSD-Ib-associated mutation P191L is shown as sticks.
(TIF)

S4 Fig Pathogenic missense mutations mapped onto human G6PT. (**A**) Details of the G6PT dimer interface, with locations of pathogenic mutations indicated and labeled. The TMs involved in subunit assembly are shown in cartoon representation, with the corresponding cryo-EM density rendered as a transparent surface. (**B**) Distribution of pathogenic missense mutations mapped onto the G6PT monomer. Mutations are represented as spheres and colored according to their reported impact on transport activities [22–25]: red, abolished activity (<15% of WT); gold, reduced activity; and gray, unknown or untested effects.
(TIF)

S5 Fig Sequence alignment of human SLC37A1–4 and GlpT. Sequence alignment of human SLC37A1–4 and their bacterial homolog GlpT. Key residues involved in substrate recognition and gate formation are highlighted in red and yellow boxes, respectively.
(TIF)

S6 Fig Sequence alignment of G6PT homologs across vertebrates. Sequence alignment of G6PT from *Homo sapiens* (Hs), *Bos taurus* (Bt), *Mus musculus* (Mm), *Lonchura striata* (Ls), *Xenopus laevis* (Xl), and *Danio rerio* (Dr). Key residues involved in substrate recognition and gate formation are highlighted in red and yellow boxes, respectively.
(TIF)

S7 Fig Predicted G6P-binding poses. (**A**) Superposition of the AF-predicted outward-open G6P–G6PT model and our experimental structure. (**B**) Binding free energies (*∆G*) for the interaction of G6P with key residues in the outward-open (left) and inward-open (right) states, as determined by MD simulations. (**C** and **D**) Structural snapshots from MD simulations of the G6P–G6PT complex in the outward-open (C) and inward-open (D) conformations, with close-up views of binding interactions between G6P and G6PT. Polar bonds are represented by dashed lines. The data underlying this figure are available in S1 Data.
(TIF)

S8 Fig Structural comparison of G6PT with independently determined models. (**A**) Structural comparison of our G6PT monomer with an independently reported apo structure in the monomeric form. This illustrates structural consistency across different experimental approaches. (**B**) Alignment of our predicted outward-open G6P–G6PT model with reported substrate-bound structures in the outward-open state. (**C**) Alignment of our predicted inward-open G6P–G6PT model with reported substrate-bound structures in the inward-open state.
(TIF)

S1 Table Cryo-EM data collection, refinement, and validation statistics. (DOCX)

S2 Table Structural mapping and functional categorization of GSD-Ib missense mutations. Missense mutations are categorized based on their structural location and putative pathogenic mechanism. For each variant, the reported protein expression level and transport activity relative to WT are provided, as compiled from previous studies [22–25]. Symbols: ↓↓ abolished activity;↓ reduced expression/activity; → expression comparable to wild-type; ↑ increased expression; ND, not determined.
(DOCX)

S1 Data Individual data used to generate Figs 2D and S7B. (XLSX)

## Abbreviations

AF-predicted: AlphaFold-predicted
CHS: cholesteryl hemisuccinate
CTD: C-terminal domain
CTF: contrast transfer function
EMDB: Electron Microscopy Data Bank
ER: endoplasmic reticulum
FSC: Fourier shell correlation
G6P: glucose-6-phosphate
G6Pases: glucose-6-phosphatases
G6PT: glucose-6-phosphate transporter
GlpT: glycerol-3-phosphate transporter
GSD-Ib: glycogen storage disease type Ib
HBSS: Hank’s balanced salt solution
ICH: intracellular helical
ipTM: interface predicted Template Modeling
LMNG: lauryl maltose neopentyl glycol
MD: molecular dynamics
MFS: major facilitator superfamily
NTD: N-terminal domain
OPA: organophosphate:phosphate antiporter
PDB: Protein Data Bank
PME: Particle Mesh Ewald
SEC: size-exclusion chromatography
SLC37: solute carrier 37
TMs: transmembrane helices
WT: wild type.
