# Supplementary figures and images for: Structures of the human glucose-6-phosphate transporter provide insights into its transport cycle and substrate recognition
Source: PLoS Biol. 2026 Mar 30;24(3):e3003731. doi: 10.1371/journal.pbio.3003731 (PMC13046256; doi:10.1371/journal.pbio.3003731)

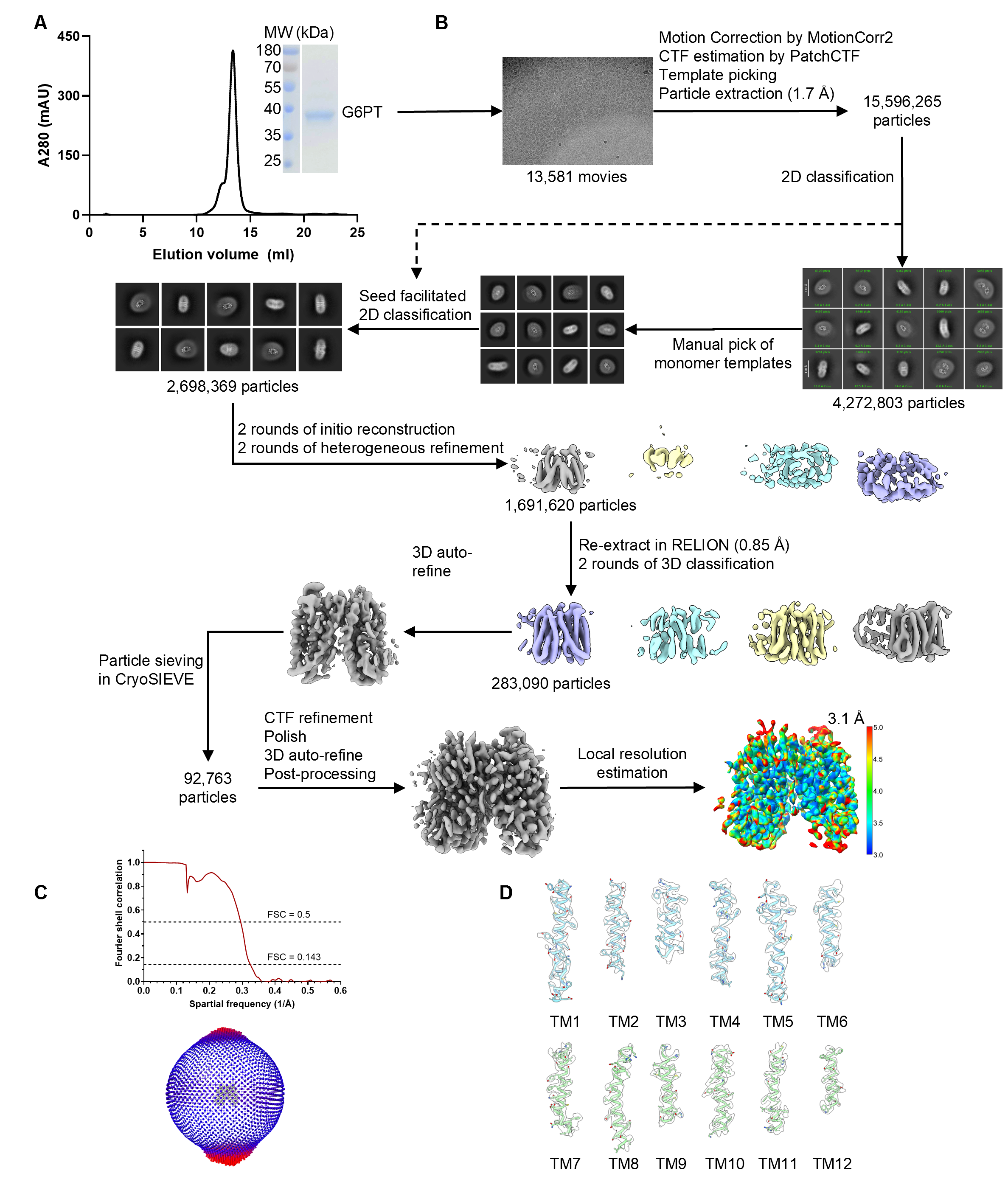

Supplement: S1 Fig — (A) Representative size-exclusion chromatography (SEC) profile and corresponding SDS-PAGE analysis of purified G6PT. (B) Cryo-EM data processing workflow for the G6PT monomer. (C) Fourier shell correlation (FSC) curve for the final reconstruction (top) and the particle orientation distribution (bottom) as evaluated by RELION 5.0. (D) Cryo-EM density maps for 12 TMs of G6PT. (TIF) [file pbio.3003731.s001.tif]

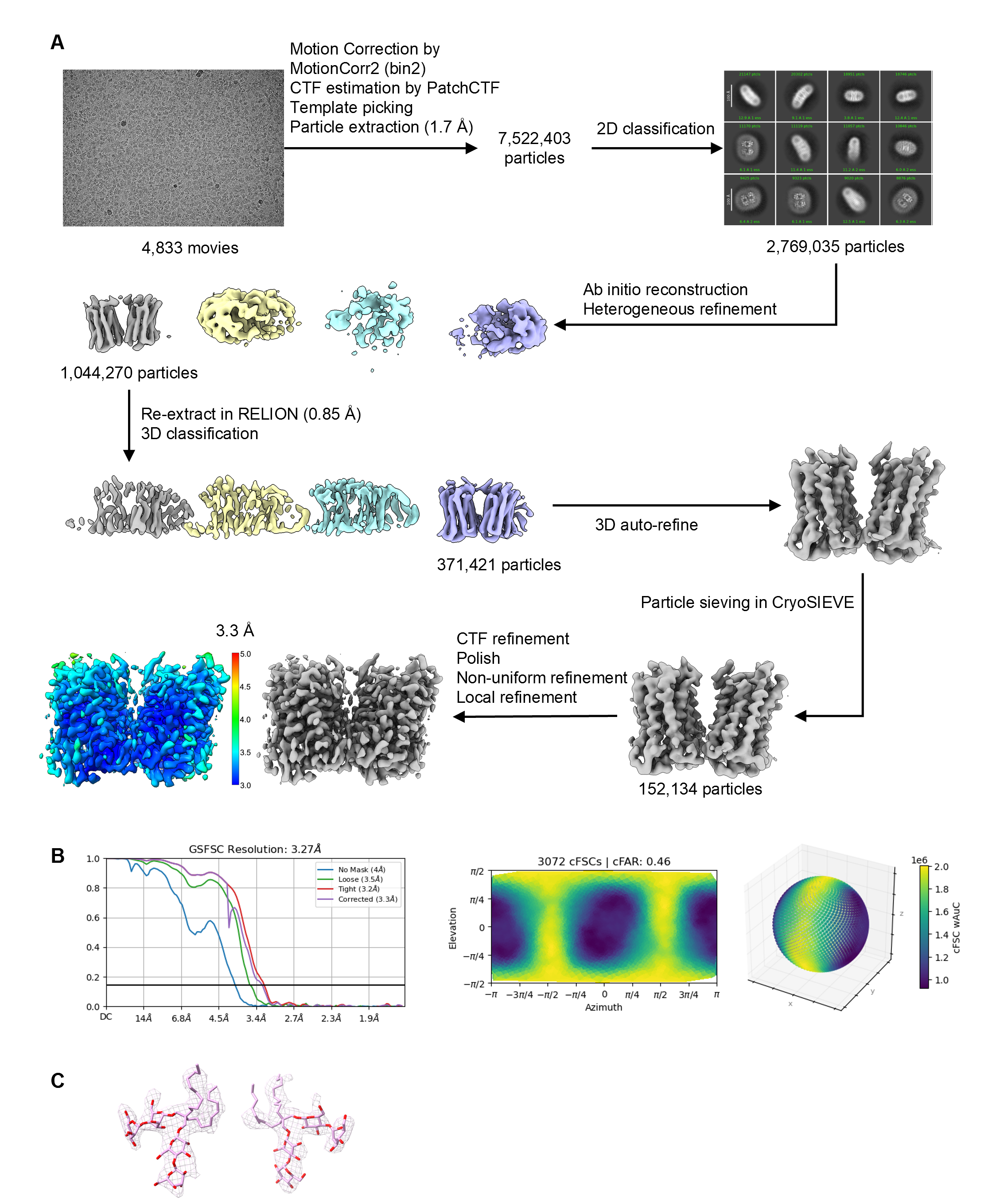

Supplement: S2 Fig — (A) Cryo-EM data processing workflow for the G6PT dimer. (B) FSC curves for the final reconstruction (left) and the particle orientation distribution (right) as evaluated by CryoSPARC. (C) Structural models of LMNG fitted into nonprotein densities at the dimer interface. (TIF) [file pbio.3003731.s002.tif]

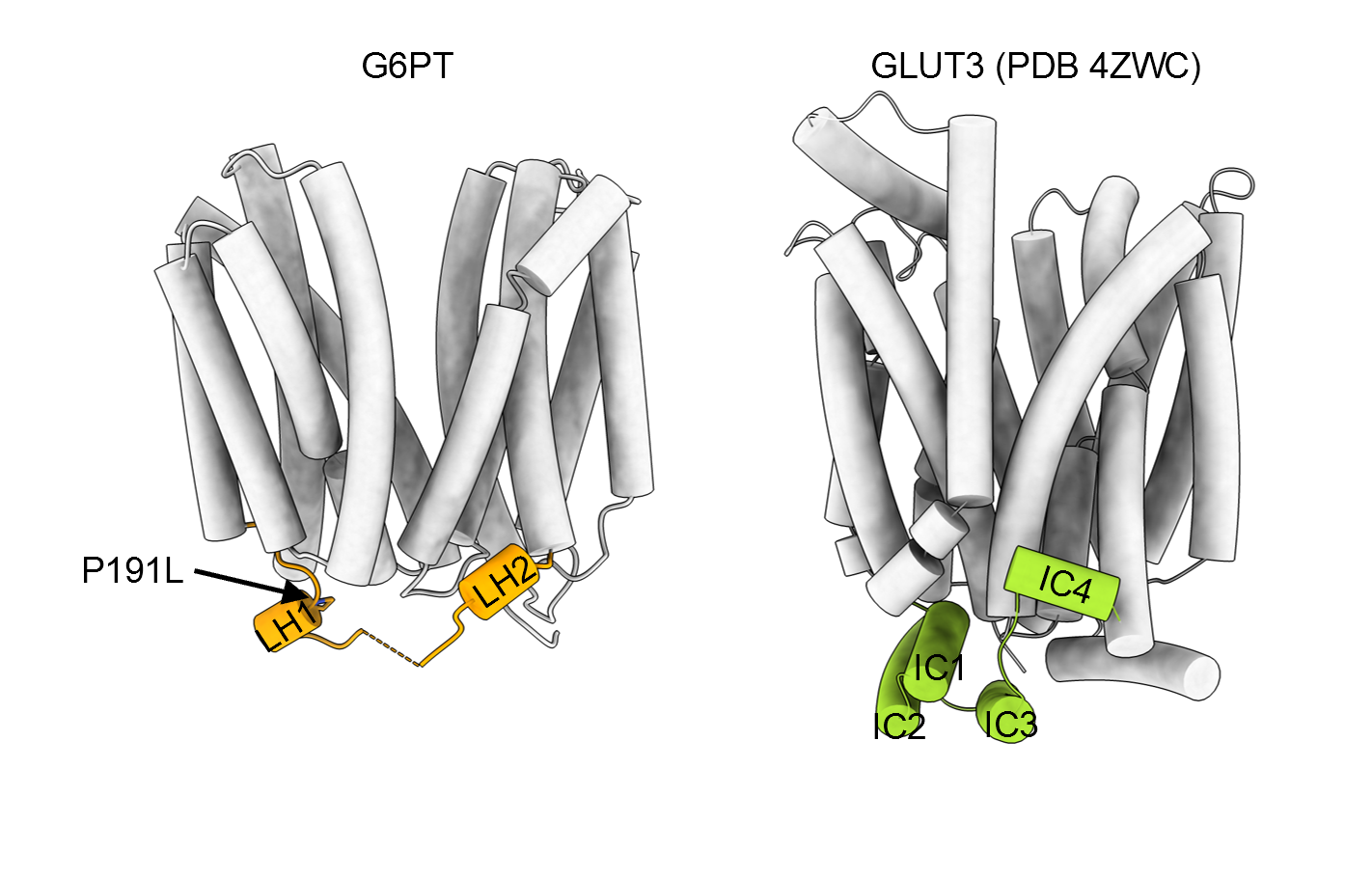

Supplement: S3 Fig — The G6PT cryo-EM structure with the L6–7 highlighted in orange and the GLUT3 structure (PDB: 4ZWC) with the intracellular helical (ICH) domain between TM6 and TM7 highlighted in green. G6PT has an L6–7 loop that lacks the triple-helix domain characteristic of GLUTs. The GSD-Ib-associated mutation P191L is shown as sticks. (TIF) [file pbio.3003731.s003.tif]

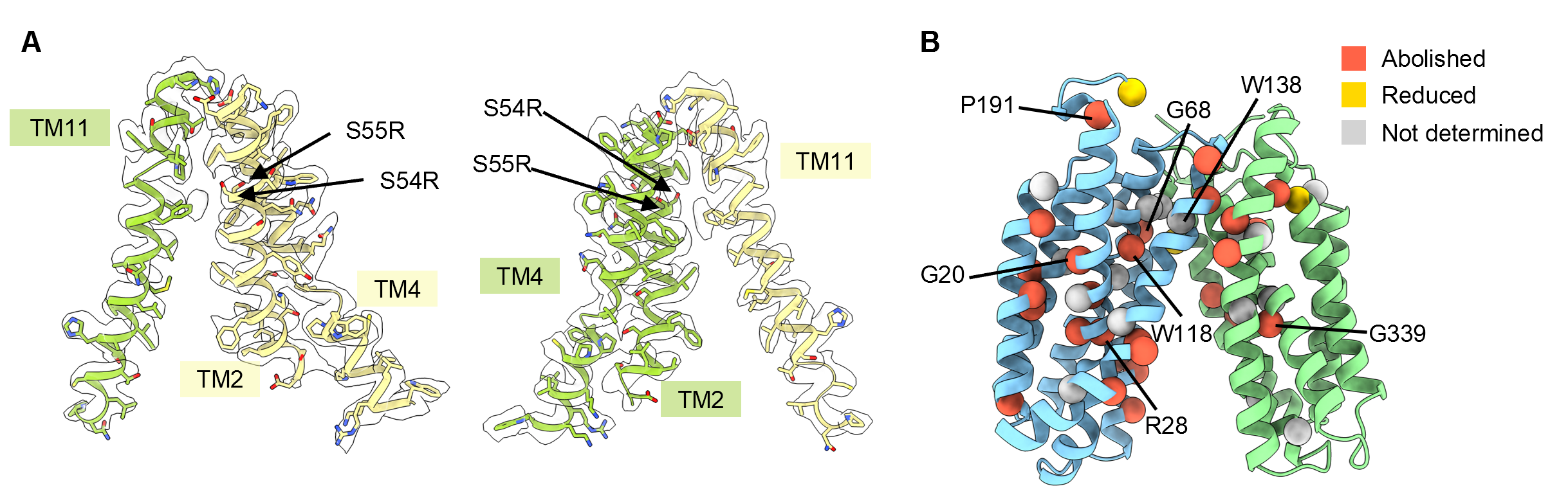

Supplement: S4 Fig — (A) Details of the G6PT dimer interface, with locations of pathogenic mutations indicated and labeled. The TMs involved in subunit assembly are shown in cartoon representation, with the corresponding cryo-EM density rendered as a transparent surface. (B) Distribution of pathogenic missense mutations mapped onto the G6PT monomer. Mutations are represented as spheres and colored according to their reported impact on transport activities [22–25]: red, abolished activity (<15% of WT); gold, reduced activity; and gray, unknown or untested effects. (TIF) [file pbio.3003731.s004.tif]

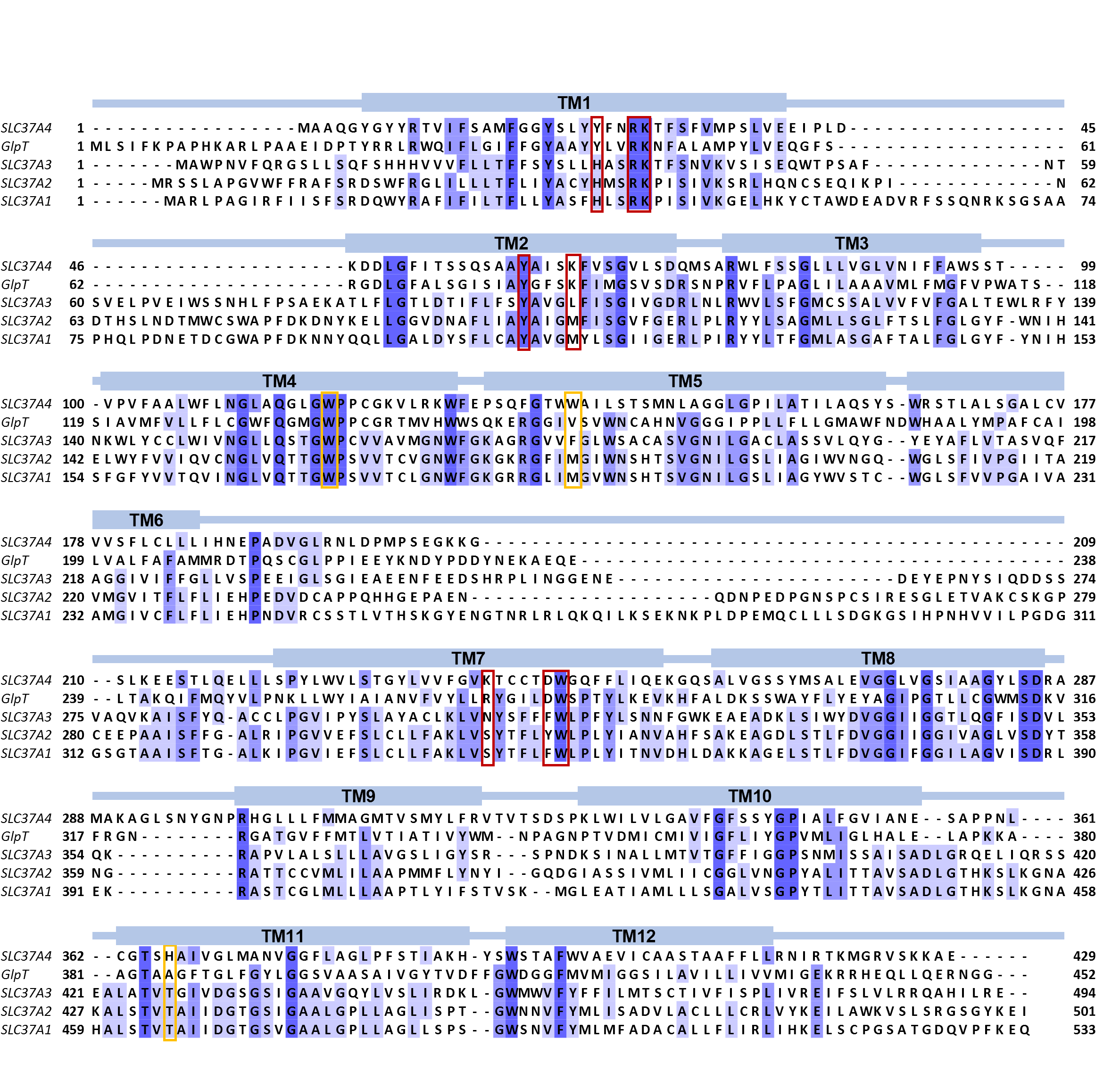

Supplement: S5 Fig — Sequence alignment of human SLC37A1–4 and their bacterial homolog GlpT. Key residues involved in substrate recognition and gate formation are highlighted in red and yellow boxes, respectively. (TIF) [file pbio.3003731.s005.tif]

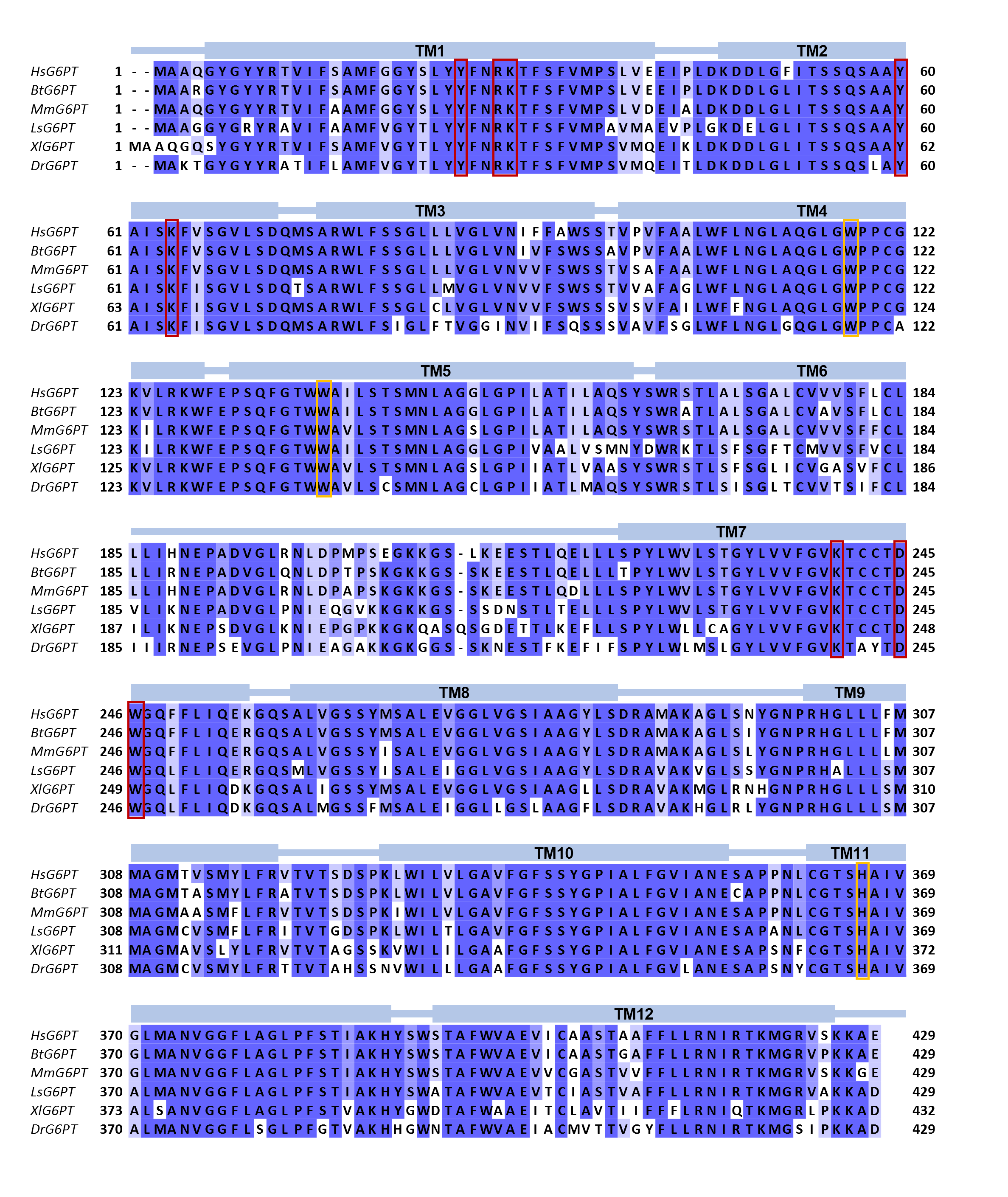

Supplement: S6 Fig — Sequence alignment of G6PT from Homo sapiens (Hs), Bos taurus (Bt), Mus musculus (Mm), Lonchura striata (Ls), Xenopus laevis (Xl), and Danio rerio (Dr). Key residues involved in substrate recognition and gate formation are highlighted in red and yellow boxes, respectively. (TIF) [file pbio.3003731.s006.tif]

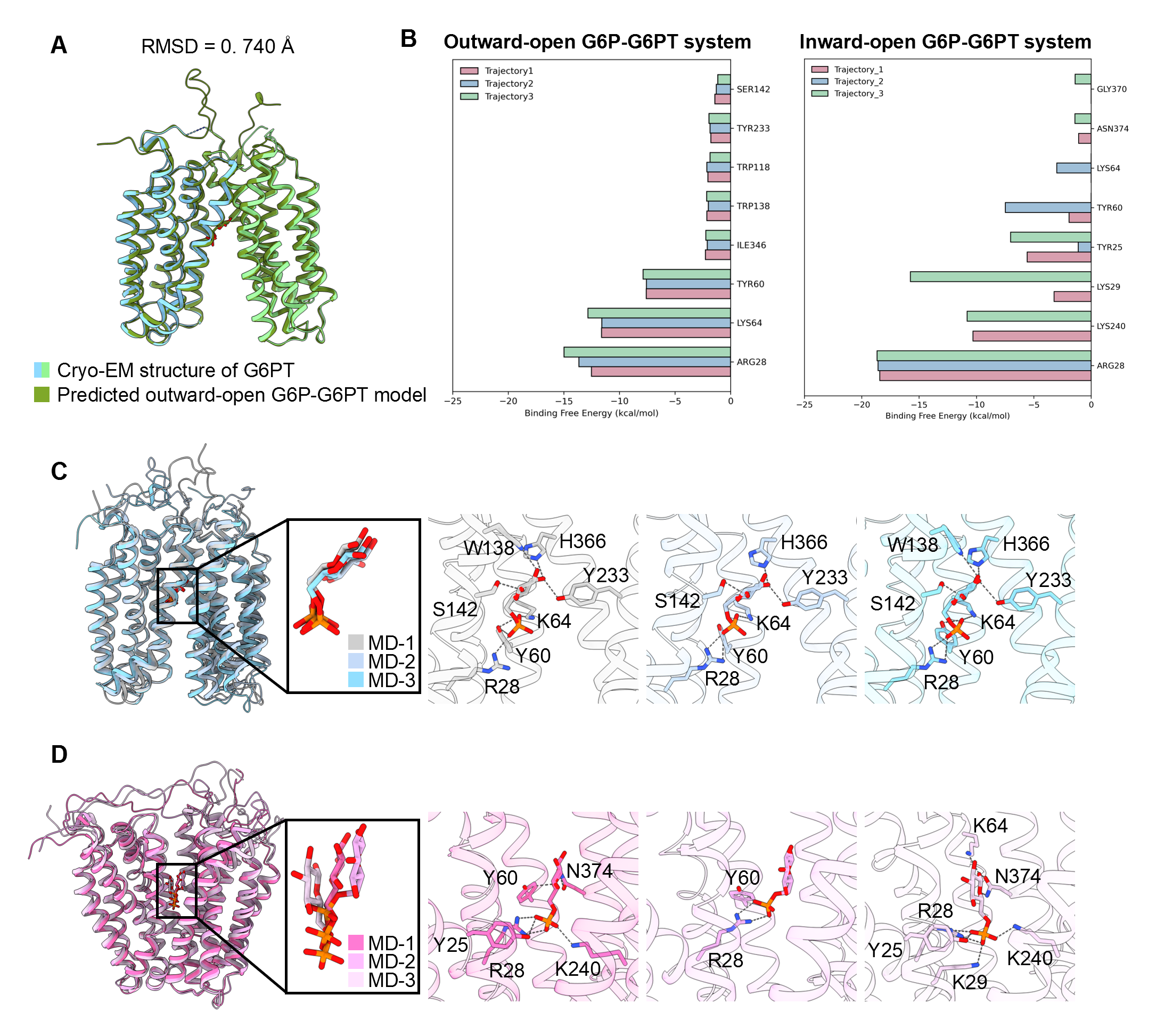

Supplement: S7 Fig — (A) Superposition of the AF-predicted outward-open G6P–G6PT model and our experimental structure. (B) Binding free energies (∆G) for the interaction of G6P with key residues in the outward-open (left) and inward-open (right) states, as determined by MD simulations. (C and D) Structural snapshots from MD simulations of the G6P–G6PT complex in the outward-open (C) and inward-open (D) conformations, with close-up views of binding interactions between G6P and G6PT. Polar bonds are represented by dashed lines. The data underlying this figure are available in S1 Data. (TIF) [file pbio.3003731.s007.tif]

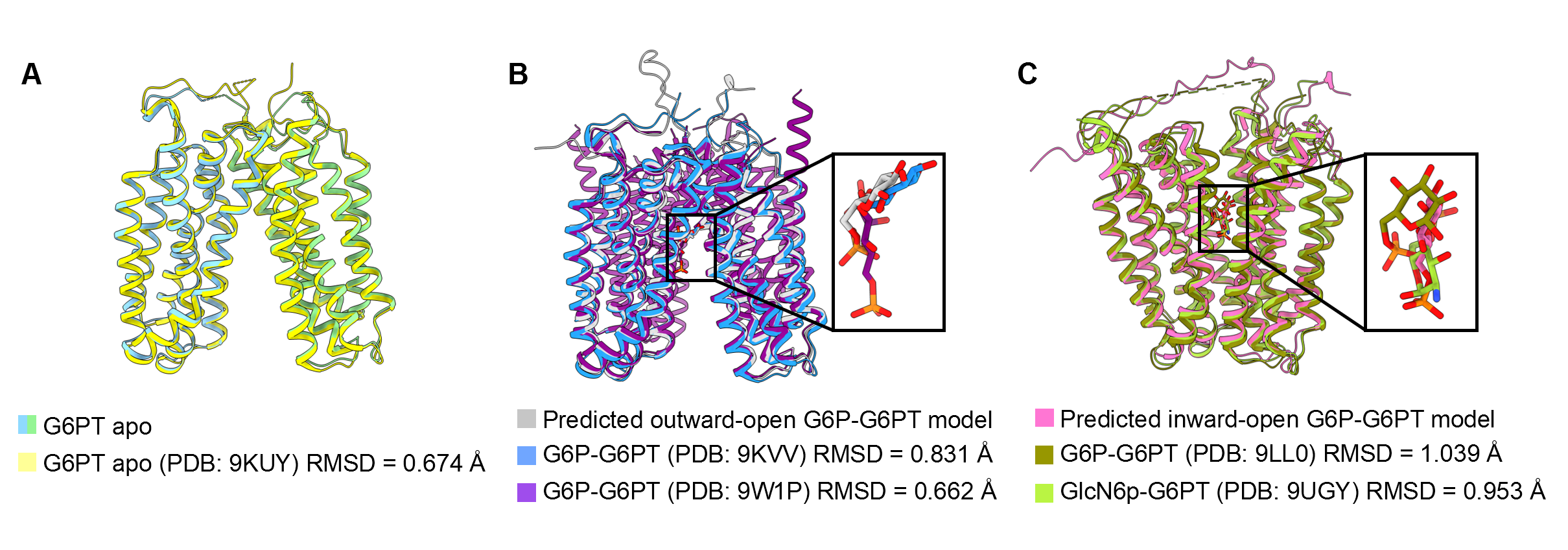

Supplement: S8 Fig — (A) Structural comparison of our G6PT monomer with an independently reported apo structure in the monomeric form. This illustrates structural consistency across different experimental approaches. (B) Alignment of our predicted outward-open G6P–G6PT model with reported substrate-bound structures in the outward-open state. (C) Alignment of our predicted inward-open G6P–G6PT model with reported substrate-bound structures in the inward-open state. (TIF) [file pbio.3003731.s008.tif]
