# Supplementary material for: Structures of the human glucose-6-phosphate transporter provide insights into its transport cycle and substrate recognition
Source: PLoS Biol. 2026 Mar 30;24(3):e3003731. doi: 10.1371/journal.pbio.3003731 (PMC13046256; doi:10.1371/journal.pbio.3003731)
Supplement: S1 Table — (DOCX) [file pbio.3003731.s009.docx]

|  | Monomer-hsG6PT | Dimer-hsG6PT |
| --- | --- | --- |
|  | (EMDB-66728) | (EMDB-66727) |
|  | (PDB-9XCB) | (PDB-9XCA) |
| **Data and processing** |  |  |
| Magnification | 105000 | 105000 |
| Voltage (kV) | 300 | 300 |
| Electron exposure (e^-^/ Å^2^) | 51.38 | 50.78 |
| Defocus range (μm) | -1.5~-2.0 | -1.5~-2.0 |
| Pixel size (Å) | 0.425 | 0.425 |
| Symmetry imposed | C1 | C1 |
| Initial particle images (no.) | 15,596,265 | 7,522,403 |
| Final particle images (no.) | 92,763 | 152,134 |
| Map resolution (Å) | 3.1 | 3.3 |
| FSC threshold | 0.143 | 0.143 |
| Map resolution range (Å) | 3.0~5.0 | 3.0~5.0 |
| **Refinement** |  |  |
| Initial model used (PDB code) | - | - |
| Model resolution (Å) | 3.3 | 3.6 |
| FSC threshold | 0.5 | 0.5 |
| Map sharpening B factor (Å^2^) | -61.1 | -134.6 |
| Model composition |  |  |
| Non-hydrogen atoms | 3095 | 6360 |
| Protein residues | 405 | 814 |
| Ligands | 0 | 2 |
| B factor (Å^2^) |  |  |
| Protein | 63.99 | 80.79 |
| Ligand | - | 20.00 |
| R.m.s deviations |  |  |
| Bond lengths (Å) | 0.003 | 0.004 |
| Bond angles (°) | 0.615 | 0.967 |
| Validation |  |  |
| MolProbity score | 1.69 | 1.65 |
| Clash score | 5.49 | 4.61 |
| Rotamers outliers (%) | 0.00 | 0.00 |
| Ramachandra plot |  |  |
| Favored | 94.26 | 93.80 |
| Allowed | 5.74 | 6.20 |
| Outliers | 0.00 | 0.00 |
