# Supplementary material for: Structures of the human glucose-6-phosphate transporter provide insights into its transport cycle and substrate recognition
Source: PLoS Biol. 2026 Mar 30;24(3):e3003731. doi: 10.1371/journal.pbio.3003731 (PMC13046256; doi:10.1371/journal.pbio.3003731)
Supplement: S2 Table — Missense mutations are categorized based on their structural location and putative pathogenic mechanism. For each variant, the reported protein expression level and transport activity relative to WT are provided, as compiled from previous studies [22–25]. Symbols: ↓↓ abolished activity;↓ reduced expression/activity; → expression comparable to wild-type; ↑ increased expression; ND, not determined. (DOCX) [file pbio.3003731.s010.docx]

| **Mutation** | **Description** | **Activity** | **Expression** |
| --- | --- | --- | --- |
| M1V | Interior of the NTD/CTD | ↓↓ | ND |
| G20D | Interior of the NTD/CTD | ↓↓ | ↓ |
| L23R | Interior of the NTD/CTD | ND | ND |
| Y24H | Interior of the NTD/CTD | ↓↓ | ↑ |
| N27K | Interior of the NTD/CTD | ↓↓ | → |
| N27S | Interior of the NTD/CTD | ND | ND |
| R28C | Translocation pathway; binding with phosphate group | ↓↓ | → |
| R28H | Translocation pathway; binding with phosphate group | ↓↓ | → |
| G50E | NTD/CTD interface | ND | ND |
| G50R | NTD/CTD interface | ↓↓ | ↓ |
| S54R | Dimer interface | ↓↓ | → |
| S55R | Dimer interface | ↓↓ | → |
| G68R | NTD/CTD interface | ↓↓ | → |
| G83E | Interior of the NTD/CTD | ND | ND |
| L85P | Interior of the NTD/CTD | ↓↓ | → |
| G88D | Interior of the NTD/CTD | ↓↓ | → |
| G115R | Interior of the NTD/CTD | ND | ND |
| W118R | Translocation pathway; formation of the cytosolic gate | ↓↓ | → |
| P120L | Interior of the NTD/CTD | ND | ND |
| G122E | NTD/CTD interface | ND | ND |
| Q133P | Interior of the NTD/CTD | ↓↓ | → |
| G135D | NTD/CTD interface | ↓↓ | → |
| W138R | NTD/CTD interface; formation of the cytosolic gate | ND | ND |
| A148V | Interior of the NTD/CTD | ND | ND |
| G149E | NTD/CTD interface | ↓↓ | → |
| G150R | NTD/CTD interface | ↓↓ | → |
| P153L | NTD/CTD interface | ↓↓ | → |
| A156V | Interior of the NTD/CTD | ND | ND |
| R166L | Interior of the NTD/CTD | ↓↓ | → |
| C176R | Interior of the NTD/CTD | ↓↓ | ↓ |
| C183R | Interior of the NTD/CTD | ↓↓ | → |
| L186P | Interior of the NTD/CTD | ND | ND |
| P191L | Protein surface or the loop region | ↓↓ | → |
| P191R | Protein surface or the loop region | ND | ND |
| N198I | Protein surface or the loop region | ↓ | ND |
| L229P | Interior of the NTD/CTD | ↓↓ | → |
| W246R | NTD/CTD interface; formation of the ER lumen gate | ND | ND |
| L251P | Interior of the NTD/CTD | ND | ND |
| G273S/D | Interior of the NTD/CTD | ND | ND |
| I278N | NTD/CTD interface | ↓↓ | ↓ |
| A280V | Interior of the NTD/CTD | ND | ND |
| G281R | NTD/CTD interface | ↓↓ | → |
| G281V | NTD/CTD interface | ND | ND |
| R300C | Interior of the NTD/CTD | ↓↓ | → |
| R300H | Interior of the NTD/CTD | ↓↓ | → |
| H301P | Interior of the NTD/CTD | ↓ | → |
| G339D | Interior of the NTD/CTD | ↓↓ | ↓ |
| G339C | Interior of the NTD/CTD | ↓↓ | ↓ |
| S342P | Interior of the NTD/CTD | ND | ND |
| L348P | Interior of the NTD/CTD | ND | ND |
| G363C | NTD/CTD interface | ND | ND |
| A367T | NTD/CTD interface | ↓ | → |
| A373D | NTD/CTD interface | ↓↓ | → |
| G376S | Interior of the NTD/CTD | ↓↓ | → |
| R415G | Protein surface or the loop region | ND | ND |
